# Is Oral Semaglutide a Good Fit for Patients After Metabolic Bariatric Surgery? A Biopharmaceutical Mechanistic Perspective

**DOI:** 10.3390/pharmaceutics18040466

**Published:** 2026-04-10

**Authors:** Almog Eliyahu Dahan, Carmil Azran, Arik Dahan

**Affiliations:** 1 Department of Clinical Pharmacology, School of Pharmacy, Faculty of Health Sciences, Ben-Gurion University of the Negev, Beer-Sheva 8410501, Israel; almogdah@post.bgu.ac.il; 2 Department of Clinical Pharmacy, Clalit Health Services, Tel-Aviv 6719709, Israel; 3 Medical Technology Department, Maccabi Health Services, Tel-Aviv 4648588, Israel; azran_c@mac.org.il; 4 School of Pharmacy, The Hebrew University of Jerusalem, Jerusalem 9112002, Israel

**Keywords:** absorption enhancer, metabolic bariatric surgery, GLP-1 RAs, SNAC, oral drug administration, peptide drugs

## Abstract

Currently, GLP-1RAs are peptide drugs, typically administered by injection due to insufficient absorption, and only one GLP-1RA, semaglutide, is available as an orally administered drug. To overcome the absorption challenges of oral peptides, this drug product contains the absorption enhancer SNAC. As the tablet is eroded in the stomach, SNAC neutralizes the acidic gastric environment, thereby protecting the semaglutide from enzymatic degradation. Then, SNAC fluidizes the stomach lipidic membrane to increase semaglutide transcellular permeability across the gastric epithelium. It is necessary to realize that the use of such a unique drug product, that relies solely on the stomach for absorption, is expected to be affected by the extreme gastric anatomy/physiology changes post-MBS. Hence, we analyzed the key mechanisms that may affect the bioavailability of oral semaglutide post-MBS. Several mechanisms appear to potentially reduce oral semaglutide absorption post-MBS, including decreased inner gastric surface area, decreased gastric contractility, and faster gastric emptying. Hence, the effectiveness of the complex formulation, that relies solely on the stomach for the SNAC activity and semaglutide absorption, may be severely hampered post-MBS; clinicians should be aware of the potential malabsorption of oral GLP-1RA post-MBS, and preferably consider subcutaneous therapy until specific pharmacokinetic/clinical data are available.

## 1. Introduction

The most effective long-term treatment available for patients with obesity is metabolic bariatric surgery (MBS). The global use of MBS has increased substantially over the past two decades. According to the International Federation for the Surgery of Obesity and Metabolic Disorders (IFSO) Global Registry, approximately 600,000 MBS procedures were performed across 37 countries and two regional registries in 2023, most commonly sleeve gastrectomy (SG) and Roux-en-Y gastric bypass (RYGB). The majority of patients undergoing MBS were female, and the most frequently reported comorbidity was type 2 diabetes [1]. However, obesity is a chronic, relapsing disease, that often requires a combination of therapeutic modalities to achieve and maintain durable weight control. Recurrence of weight gain, reappearance or worsening of obesity-related comorbidities may require close monitoring and additional interventions [2].

Despite the substantial benefits of MBS, approximately 20–30% experience insufficient weight loss or significant weight regain after MBS, depending on the surgical procedure [3,4]. Furthermore, diabetes relapse has been reported in 13–20% of individuals who initially achieved remission following MBS within 10 years [5]. An additional complication of MBS is that the altered gastrointestinal (GI) tract anatomy/physiology may significantly impact the pharmacokinetics and pharmacodynamics of various orally administered medications [6].

In recent years, highly potent medications for obesity and diabetes management, glucagon-like peptide-1 receptor agonists (GLP-1RAs), have become available, among other therapies. This is particularly relevant for patients experiencing weight regain, diabetes relapse, or poor glycemic control following MBS. Indeed, it was recently reported that one in seven bariatric patients is prescribed GLP-1RAs, and their use among patients who underwent MBS is steadily increasing over time [3].

The oral route is by far the preferred and most convenient way of drug administration [7,8]. Currently, GLP-1RAs are peptide drugs, typically administered by injection due to their low oral bioavailability, attributable to degradation in the GI milieu by proteolytic enzymes and insufficient permeability across the GI membrane. To date, only one GLP-1RA, semaglutide, is available as an orally administered drug product [9]. Until recently, oral semaglutide was available only for patients with type 2 diabetes under the name Rybelsus^®^. In December 2025, the U.S. Food and Drug Administration (FDA) approved Wegovy^®^ pill, the first (and currently only) oral GLP-1 RA for weight loss treatment [10]. The currently available oral semaglutide formulations (e.g., Rybelsus^®^ and oral Wegovy^®^) use sodium N-[8-(2-hydroxybenzoyl) aminocaprylate] (SNAC) absorption technology.

A systematic review and meta-analysis suggest that GLP-1RAs are both safe and effective in post-MBS patients, at least for the short-term [11]. However, despite evidence supporting their clinical utility for weight regain and glycemic control, data regarding the absorption and pharmacokinetics of orally administered GLP-1RA, semaglutide, following MBS remain scarce.

In this perspective, we present various mechanisms by which bariatric procedures may influence oral drug absorption and analyze the mechanisms that may contribute to alterations in oral semaglutide absorption following MBS.

## 2. The Unique Absorption Mechanism of Current Semaglutide Pill

**Figure 1:**
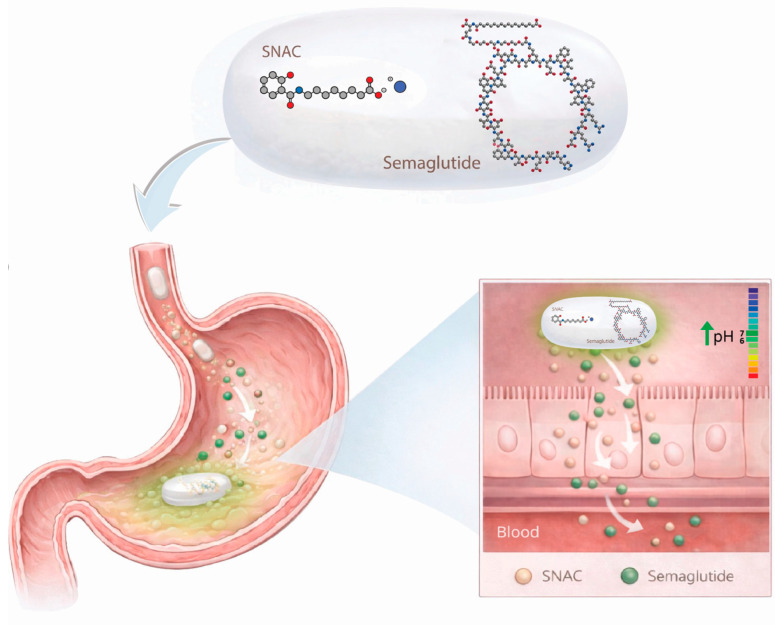
Structure of semaglutide and SNAC in an oral pill formulation, followed by the mechanism by which SNAC impacts semaglutide absorption in the stomach. SNAC, sodium N-[8-(2-hydroxybenzoyl) aminocaprylate]. Atoms are represented by color: carbon, gray; oxygen, red; nitrogen, blue. Molecular structures source: dreamstime.com; software used for figure preparation: Adobe Illustrator (CC 2019).

The development of an oral GLP-1RA formulation, such as semaglutide, presents a challenge, as absorption occurs in the stomach. Achieving sufficient systemic exposure of peptide-based drugs following oral administration is difficult due to the acidic gastric environment, the presence of proteolytic enzymes, and the limited permeability of peptides through the GI epithelium. To overcome the absorption challenges of oral peptides, this drug product contains the absorption enhancer SNAC. As the tablet is eroded in the stomach, SNAC neutralizes the acidic gastric environment, increasing the local pH to interfere with pepsinogen activation, thereby protecting semaglutide from enzymatic degradation, as well as promoting monomerization. In addition, SNAC fluidizes the stomach lipidic membrane to increase semaglutide transcellular permeability across the gastric epithelium (Figure 1). To avoid decreased absorption, patients are instructed to administer the tablet once daily on an empty stomach, with a sip of water (up to half a glass of water, equivalent to 120 mL), and wait at least 30 min before eating or drinking or taking other oral medications [8,12,13].

## 3. Impact of MBS Type on Drug Absorption

Pharmacotherapy following MBS is a major and complex treatment challenge. The substantially altered GI anatomy and physiology after the surgery may greatly impact the pharmacokinetics of orally administered drugs, with potentially significant clinical implications. The complex process of drug absorption involves multiple stages, and many of them may be affected by the surgery. These changes include alterations in gastric pH, reduced gastric volume, changes in GI transit time, decreased absorptive surface area, and modifications in metabolizing enzymes and transporters [14]. Previous studies have demonstrated that such physiological and anatomical alterations may significantly influence drug absorption, and systemic drug exposure from different pharmacological classes [15]. Consequently, drug absorption and bioavailability may decrease, increase, or remain unchanged following MBS, depending on the specific drug, the type of bariatric procedure performed and also patient-related factors.

Reduced drug absorption following MBS has been reported for several medications. Among anticonvulsants, decreased drug exposure has been described for phenytoin [16,17], lamotrigine [18] and valproic acid [19], leading to subtherapeutic serum levels and recurrence of seizures. Similar effects have been reported for immunosuppressants such as cyclosporin [20,21], and mycophenolic acid [22], often requiring dose adjustments. Reduced drug exposure has also been observed with antidepressants including escitalopram [23], citalopram, sertraline, and mirtazapine [24], as well as certain anticancer drugs such as tamoxifen, which in some cases require dose escalation, discontinuation of therapy, or switching to alternative treatment [25,26].

Conversely, increased drug exposure following MBS has also been described. Lithium toxicity has been reported due to increased drug solubility and decreased renal lithium excretion associated with reduced fluid intake [27,28]. Increased systemic exposure has also been observed for metformin [29]. Similarly, pharmacokinetic studies have demonstrated increased bioavailability of atorvastatin several weeks after bypass surgery in the majority of patients, likely due to reduced first-pass metabolism [30,31]. The wide variability in drug levels observed after MBS, ranging from subtherapeutic concentrations to potential toxicity, underscores the complexity of pharmacotherapy management in patients undergoing bariatric surgery.

**Figure 2:**
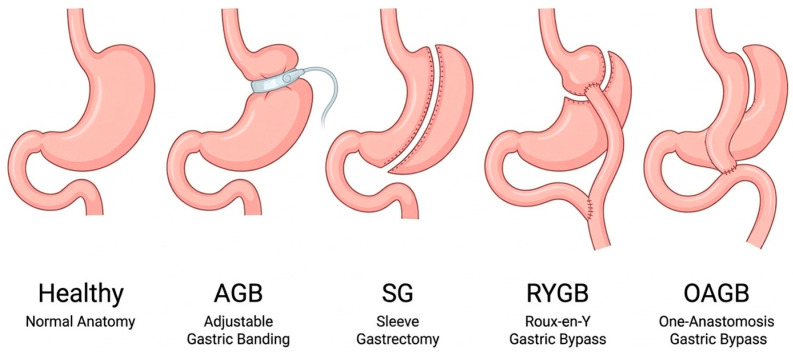
Schematic Illustration of Metabolic Bariatric Surgery Procedures. The figure presents the anatomical configuration of the healthy stomach (normal anatomy) and four bariatric procedures: adjustable gastric banding (AGB), sleeve gastrectomy (SG), Roux-en-Y gastric bypass (RYGB), and one-anastomosis gastric bypass (OAGB).

MBS are broadly classified into three categories: restrictive procedures, malabsorptive and combined procedures. Restrictive surgeries (e.g., SG, adjustable gastric banding (AGB)) primarily reduce the size of the stomach, limiting food intake. SG reduces the volume of the stomach by a longitudinal resection of its greater curvature part and AGB involves the implantation of an adjustable band around the upper part of the stomach. Due to insufficient weight loss and higher re-operation rate, the use of AGB has decreased significantly [32]. Combined surgeries (e.g., RYGB, one-anastomosis gastric bypass (OAGB)) reduce gastric volume with bypass portions of the small intestine. Malabsorptive surgeries (e.g., jejunoileal bypass), isolate the proximal jejunum to distal ileum segment, resulting in bypass of the vast majority of the small intestine. Due to concerns related to nutrient absorption, the use of this procedure is rare [19]. A schematic illustration of the MBS discussed in this section is presented in Figure 2.

Notably, restrictive procedures can be expected to have fewer pharmacokinetic effects than malabsorptive or combined procedures [33]. Therefore, the exact type of MBS performed directly influences the potential for alteration in the pharmacokinetics of orally administered medications, and hence this is a major factor when analyzing the drugs taken by a specific bariatric patient [34]. In the context of this article, although significant differences exist among bariatric procedures, all types of MBS drastically affect the stomach. Therefore, each procedure is expected to influence the absorption of oral semaglutide.

## 4. Potential Mechanisms Affecting Oral Drug Absorption Following MBS

The process of drug absorption from the GI tract is complex and involves multiple stages; each one may be affected following MBS. After swallowing, a solid immediate-release (IR) oral drug product must disintegrate into small particles to enable drug release in the stomach. Because all metabolic bariatric procedures significantly reduce stomach size (by approximately 80%) and contractility, gastric mixing with the stomach content is impaired, which may result in insufficient tablet disintegration [6].

One solution to insufficient disintegration is the use of liquid oral dosage forms (e.g., syrup), in which the drug is already dissolved. In cases where only solid dosage forms are available, patients should be instructed to crush tablets or open capsules and disperse the contents in liquid prior to ingestion (according to package insert or available company data) [6]. Roskam-Kwint et al. [35] reported that crushing the fixed-dose combination of dolutegravir/abacavir/lamivudine resulted in increased dolutegravir exposure, with a 26% increase in AUC and a 30% increase in C_max_.

Once the drug is released from the drug product, it needs to dissolve in the GI fluids, which is a prerequisite for subsequent absorption. For drugs with borderline solubility, the reduced stomach volume, decreased fluid intake, and diminished contractility may result in limited and inadequate drug dissolution.

The dissolution process of many drugs is pH-dependent, and gastric pH increases significantly after MBS due to a reduction in acid-producing parietal cells, resulting in higher pH levels compared with preoperative values (pH~2). Postoperative measurements demonstrate significantly elevated pH levels, particularly among patients undergoing OAGB (pH~6.5), compared with those undergoing SG (pH~5) [36]. As a result, the solubility of basic drugs may be hampered following MBS, while for acidic drugs the solubility may increase [6]. In addition, many patients receive acid-reducing medications (e.g., PPIs or H2 blockers) after MBS to reduce the risk of marginal ulcers following bypass procedures and to manage or prevent acid related complications such as gastroesophageal reflux disease (GERD) [37,38,39], which also contributes to an increase in gastric pH. Several drugs exhibit pH-dependent solubility and dissolution, and their absorption may therefore be affected by the increased gastric pH following MBS. For example, lamotrigine is a marginally soluble weak base drug. Decreased lamotrigine solubility with increasing pH (from 1.37 ± 0.09 at pH = 1 to 0.22 ± 0.03 mg/mL at pH = 7) was observed. Dissolution studies showed that only the lowest dose of lamotrigine (25 mg) fully dissolved in the post-surgery stomach conditions, while at higher doses, tablet dissolution was impaired [40]. Similarly, loratadine, a weakly basic antihistamine, exhibits pH-dependent solubility with decreased solubility at higher pH. An experimental study simulating post-bariatric gastric conditions demonstrated severely impaired loratadine dissolution, dropping from complete dissolution before surgery to only 3% and 1% after SG and OAGB, respectively [41].

In another dissolution study, the entire dipyridamole dose dissolved in the acidic, non-operated gastric conditions, whereas in post-MBS condition (either SG or OAGB) the dissolution was solubility-limited, and only a very low percentage of the drug was dissolved [36]. Indeed, this is also one of the mechanisms proposed for changing levothyroxine pharmacokinetics after MBS [42]. Experimental solubility data of levothyroxine sodium showed a decrease across the physiologically relevant pH range of 1 to 6, with solubility values of 10 µg/mL at pH 1 and approximately 0.25 µg/mL at pH 3 to 6 [43].

Of note, this mechanism may also be relevant to acid compounds. The solubility/dissolution of acidic drugs may increase, potentially leading to increased absorption and bioavailability. This has been proposed as one of the mechanisms underlying the increased blood levels of anti-HIV drug dolutegravir after SG, as the drug becomes more soluble in the elevated gastric pH environment [44]. Furthermore, elevated gastric pH after MBS may alter the absorption of lithium, administered as a carbonate salt. In a less acidic stomach environment, carbonate is in its deprotonated form, promoting enhanced dissolution of lithium carbonate, and increasing the risk of lithium toxicity [28,45,46,47,48].

After gastric emptying from the stomach, the drug is then transferred into the duodenum. Lipophilic drugs may require bile and pancreatic secretions for solubility/dissolution. However, in patients that undergo malabsorptive MBS (e.g., RYGB), upper small intestinal segments are bypassed, and these secretions are diverted to lower segments, which may hamper drug solubilization.

When the drug is dissolved in the intestinal fluids, it is available for permeation across the gut membrane into the enterocytes. Many drugs require the entire small intestinal length, surface area, and transit time to achieve adequate absorption, and since bypass procedures reduce all three parameters, absorption may be impaired.

This permeation process may be passive, based on simple diffusion across the enterocyte, or alternatively, may involve active transport by carrier proteins. The expression of these transporters may be region-dependent, and hence, malabsorptive procedures that bypass a significant portion of the small intestine may change the exposure of drugs to relevant transporters, thereby changing their absorption profile.

Similarly, the expression of metabolic enzymes along the GI tract may vary along the small intestine, and bypassing the upper intestine by malabsorptive procedures may change the fraction of dose that escapes pre-systemic intestinal metabolism; CYP450-3A4 is such an enzyme, which is highly expressed at the upper small intestine and decreases with progression to more distal areas. Malabsorptive procedures (e.g., RYGB) bypass the upper small intestine; therefore, drugs that are substrates of this enzyme may have a higher fraction of administered dose reaching the bloodstream, potentially resulting in increased bioavailability following these procedures. It should be noted, however, that GI adaptation occurs over time, and thus alterations in drug absorption during the first months after MBS may be transient [6]. For example, the systemic exposure of atorvastatin increases during the first weeks after bypass surgery and renormalizes over time to preoperational values due to an intestinal adaptation [31].

Subsequently, drug molecules pass through the liver before reaching the systemic circulation and may undergo first pass pre-systemic hepatic metabolism. This process may also be influenced by MBS, as rapid weight loss is associated with a reduction in liver size, which leads to decreased hepatic metabolism and, consequently, increased drug bioavailability. In addition, the significant loss of adipose tissue following MBS may alter drug distribution and overall pharmacokinetic profiles [49,50].

Another important metabolic pathway is glucuronidation. This process is catalyzed by UDP-glucuronosyltransferases (UGTs), which are highly expressed in the liver and play a major role in hepatic clearance. Obesity is one of several factors that have been reported to be associated with increased UGT activity [51,52]. Following MBS, patients experience substantial weight loss, accompanied by a reduction in adipose tissue, which is rich in glucuronidation enzymes, as well as a reduction in liver size. Previous PK studies have shown that the clearance of several drugs, including oxazepam and lorazepam, both substrates of various UGTs, is increased in patients with obesity [53].

In another study, the systemic bioavailability of orally administered paracetamol was shown to be increased after SG compared with preoperative levels. Patients undergoing SG experience significant weight loss, resulting in a reduction in adipose tissue and glucuronidation enzyme capacity, which may contribute to higher post-surgery paracetamol plasma concentrations [54]. Finally, renal function is altered in patients with obesity, and after substantial weight changes; the limited fluid intake after MBS can further impair renal function, with potentially reduced excretion and increased overall exposure of relevant drugs [14].

Importantly, among orally administered drugs, semaglutide represents a unique formulation, as its absorption occurs in the stomach and is facilitated by the absorption enhancer SNAC. Therefore, the substantial anatomical and physiological changes following MBS may have complex and potentially opposing effects on oral semaglutide absorption. The reduced gastric surface area and altered gastric emptying may impair drug absorption. Conversely, the increase in gastric pH observed after several bariatric procedures may theoretically favor oral semaglutide absorption. Several additional drugs, including etoricoxib [55], sildenafil [56], and certain antifungal agents [57], have also been shown to exhibit impaired dissolution/solubility due to alterations in gastric anatomy and physiology, highlighting the importance of gastric physiology in determining drug absorption and bioavailability. The specific mechanisms that may influence oral semaglutide absorption after MBS are discussed in the following section.

## 5. Analysis of the Impact of MBS on Oral Semaglutide Absorption

It is prudent to realize that the use of such a unique drug product that relies solely on the stomach for absorption, is expected to be affected by the extreme gastric anatomy/physiology changes post-surgery. Currently, no clinical studies have specifically evaluated the pharmacokinetics of oral semaglutide following MBS. Therefore, the available evidence is largely mechanistic. Hence, we analyzed the key mechanisms that may affect the bioavailability of oral semaglutide after MBS (Table 1).

The first parameter that is drastically altered post-MBS is the gastric mass. In SG, ~80% of the stomach is removed, and even more than that in gastric bypass procedures. This results in a parallel reduction in stomach inner surface area, which is the only absorption site of oral semaglutide, so the SNAC/semaglutide activity may be severely hampered. Gastric contractility may also be hampered post-surgery due to the stomach resection, limiting the process of tablet erosion. Moreover, while GLP-1RAs delay gastric emptying pharmacologically, gastric emptying becomes faster post-MBS, reducing the time available for semaglutide absorption in the stomach. These mechanisms may lead to insufficient absorption and bioavailability of oral semaglutide after MBS.

The stomach mass resection also decreases the amount of gastric parietal cells that secrete hydrochloric acid, increasing the fasted-state gastric pH to ~5–7 [14,36]. This increased pH was shown to influence the pharmacokinetics of various drugs. However, since SNAC itself induces a localized gastric pH elevation, this mechanism is not expected to affect oral semaglutide absorption. Indeed, Baekdal et al. [62] reported no influence of omeprazole on oral semaglutide exposure.

Semaglutide has an isoelectric point (pI) of 5.4 and exhibits low solubility within the pH range of 2–6. Molecules carry no net charge at pI, which may promote semaglutide aggregation and potentially impair dissolution and stability [63]. Indeed, an experimental stability study revealed a higher extent of degradation of semaglutide at pH 4.5–5.5 [64]. Gastric pH values approaching this pH range following MBS may theoretically influence dissolution and physicochemical stability of semaglutide in the gastric environment. Nevertheless, current oral semaglutide tablets are co-formulated with SNAC, which locally increases gastric pH and facilitates semaglutide stability and absorption.

Additional anatomy/physiology changes post-MBS are related to the bypass of proximal intestinal segments, but these are less relevant to oral semaglutide which is absorbed solely in the stomach. It should be noted that the time elapsed since surgery and stomach adaptation processes are important factors that may further complicate predicting the absorption of oral semaglutide post-MBS.

## 6. Conclusions and Future Directions

Several mechanisms appear to potentially reduce oral semaglutide absorption post-MBS compared to non-operated patients. The effectiveness of the complex formulation that relies solely on the stomach for the SNAC activity and semaglutide absorption may be significantly impaired after the major gastric changes post-surgery; and since the summary of product characteristics for oral semaglutide declares that there is no therapeutic experience in bariatric patients [65], clinicians should be aware of the potential malabsorption of oral GLP-1RA post-MBS, and preferably consider subcutaneous therapy until specific pharmacokinetic/clinical data are available. In bariatric patients already treated with Rybelsus^®^ or oral Wegovy^®^, HbA1C, fasting blood glucose, and BMI require close monitoring. Further laboratory and clinical research are needed to explore the efficacy of using oral semaglutide in patients undergoing MBS. Noteworthy, an oral non-peptide small-molecule GLP-1 RA, orforglipron, was recently (1 April 2026) approved by the FDA under the Commissioner’s National Priority Voucher (CNPV) pilot program. It can be taken at any time of the day and without food or water restrictions. Such a drug may serve as an oral GLP-1RA treatment option for patients post-MBS.

## Figures and Tables

**Table 1:** Summary of mechanisms affecting oral semaglutide exposure after bariatric surgery (gastric bypass or SG) compared to the non-surgical condition.

Proposed Mechanism	Non-Surgical	SG	Gastric Bypass
Gastric volume (fed state)	~1000–1500 mL [58]	~140–210 mL [59]	~30 mL [60,61]
Stomach surface area	Normal	↓	↓↓
Gastric contractility and tablet erosion	Normal gastric motility	↓	↓↓
Gastric emptying	↓ (GLP-1RAs delay gastric emptying)	↑	↑↑
Gastric pH	1–2	~5 [36]	~6.5 [36]
Preferred route of administration of GLP-1 RA	Oral or SC	SC	SC

SC = subcutaneous.

## Data Availability

No datasets were generated or analyzed during the current study.
